# Does a bursary scheme for students in low- to middle-income countries influence outcomes in a master’s programme in Public Health?

**DOI:** 10.15694/mep.2019.000122.1

**Published:** 2019-06-04

**Authors:** Pasipanodya Ian Machingura, Saran Shantikumar, Olukemi Solabomi Babalola, Richard F Heller

**Affiliations:** 1 University of Zimbabwe; 2 University of Warwick; 3 Center for Health Policy; 4 People's Open Access Education Initiative (Peoples-uni)

**Keywords:** Scholarships, Online Education, Public Health, Low- to Middle-Income, Master’s

## Abstract

This article was migrated. The article was marked as recommended.

Introduction

The People’s Open Access Education Initiative (Peoples-uni) provides online education for health professionals in Public Health at the master’s level. Although fees are low due to the use of volunteers and Open Educational Resources, a bursary scheme is provided to waive all or some of the fees. This study tests the hypothesis that student outcomes of completing and passing modules are higher among those given a bursary than others.

Methods

Data were retrieved for all students enrolling between 2009-2017, including demographics and module outcomes, where available. Multivariable logistic regression was used to identify factors associated with a successful bursary application, as well as to elicit whether a successful bursary application was associated with ever completing, or ever passing, a module.

Results

Data were obtained from 1499 students. Of these, 624 (42%) had ever completed a module, and 513 (34%) had ever passed a module. 503 students (34%) had applied for a bursary, of whom 285 (57%) were successful. After adjusting for demographic variables, employment status and education level, students who were given a bursary were more likely to ever pass a module (adjusted odds ratio [aOR] 2.3, 95% CI 1.7,3.3), as were those who applied for a bursary but were unsuccessful (aOR 1.9, 95% CI 1.3,2.8), compared with students who had not applied for a bursary. Similar results were obtained for the outcome of completing a module.

Conclusions

Students who were successful in gaining a bursary, as well as those who were not but still able to enroll, were more likely to complete or pass a module than those who did not apply. These results point to the success of the bursary scheme and give us confidence to continue to offer bursaries, in order to sustain the mission of improving population health through capacity building in low resource settings.

## Introduction

Worldwide, there is an emphasis on education, thus the UN Sustainable Development Goals (SDGs) includes a standalone goal for education (Goal 4) “ *ensure inclusive and quality education for all and promote lifelong learning* *opportunities for all*” ( Vladimirova and Le Blanc, 2016). The changing global Public Health context has also brought about an emphasis on education, which requires corresponding changes in Public Health education, particularly for those in low- and middle-income countries ( Heller *et al.*, 2007). Complex Public Health responses require highly skilled Public Health personnel with expertise and knowledge to contribute to strengthening of health systems for optimal service provision ( Zwanikken, Alexander and Scherpbier, 2016). Training programmes such as the Master of Public Health are viewed as essential contributors in equipping healthcare workers to meet the demands of health systems ( Zwanikken *et al.*, 2013; Dlungwane *et al.*, 2017).

The People’s Open Access Education Initiative (Peoples-uni) has the mission “ *To contribute to improvements in the health of populations in low- to middle-income countries by building Public Health capacity via e-learning at very low cost*”( Peoples-uni, 2008). Individual master’s level modules have been offered online to health professionals since 2008 and provide academic credit towards a Master of Public Health degree ( Heller, Strobl and Madhok, 2019). An independent evaluation ( Sridharan *et al.*, 2018) has suggested that Peoples-uni provides online education in line with Sustainable Development Goal 4 and that credible and positive outcomes can be achieved amongst graduates of the programme.

Costs are kept low through our stated objective to “ *Utilise a ‘social model’ of capacity building, with volunteer academic and support staff and Open Educational Resources.*.”( Peoples-uni, 2008; Heller RF, 2009). Students enrol in individual modules each semester, and can then apply to join the master’s programme.

In order to support the Peoples-uni infrastructure, a small fee is charged to the students. The fee per module was GBP40 (now GBP50), adding to GBP320 (now GBP500) for the master’s level award. Higher amounts were required for the master’s degree offered by a partner university based on the same set of studies (GBP1500). However, many of the potential students are unable to pay the fees charged due to the settings in which they live and work. In order not to deny such people the opportunity to access this educational opportunity, a bursary system was instigated. However, whether outcomes are influenced by the bursary scheme, which waives all or some of the fees, is unclear.

This paper tests the hypothesis that student outcomes of completing and passing study modules are better among those given a bursary than others.

## Methods

Enrolment is semester by semester with an application form to be completed for entry to either one or two course modules each semester. The application form includes the following questions: “ *If you cannot afford the fees, we may be able to assist in approved cases. If you would like to apply for a reduction or waiver of the fees.., please provide details on: 1. What is your current employment AND monthly gross income; 2. What is the reason you are unable to pay the fees; 3. Whether you are able to pay a portion of the fees and if so how much; and 4. How you plan to use the skills/qualifications you will gain from Peoples-uni for the health of the population (up to 150 words).*” A small group independent of the course educational administrative processes, including three experienced tutors with knowledge of international living costs and the Chair of the Trustees (who adjudicated any differences in recommendations), assessed the applications and decided to award a full bursary, a partial bursary or to reject the application. For returning students there was also information on student performance in previous modules which could be taken into account in cases of disagreement. There was no fixed number of bursaries, but a notional ceiling of bursary awards to 20% of the semester enrolments was applied.

Data from all Peoples-uni registrants since the first semester in 2009 to the second semester in 2017, in whom data were available, were retrieved to obtain: (1) demographic (age, sex, country of origin), education and employment details; (2) information on whether modules enrolled on were completed (i.e. all assignments submitted) and passed; and (3) whether students had registered for the master’s programme in Public Health. Students who pass at least two modules are eligible to enrol in the master’s programme, although all modules are delivered and assessed at the master’s level. Graduation from the master’s programme was not explored as analyses of completion and passing were assessed at the module level.

### Statistical Analysis

Since students would take a variable number of modules over the course of their study, and the bursary information was obtained separately for each semester, ‘the student’ was the unit of enquiry. The characteristics of students who have (1) ever received a bursary, (2) ever applied for but not received a bursary, and (3) never applied for a bursary were compared using descriptive statistics. In those students who ever applied for a bursary, we examined the factors associated with ever being successful in the application. The two primary outcomes examined were having ever completed, and ever having passed, a module. Categorical variables were analysed using a chi-squared test, and continuous, normally distributed variables were compared using a two-tailed Student t-test. The association between student characteristics and binary outcomes were tested using multivariable logistic regression. Multiple imputation was used to substitute missing data under the assumption that these were missing at random. The results of regression are presented as odds ratios (OR), or adjusted odds ratios (aOR), with 95% confidence intervals. A *p* value < 0.05 was considered statistically significant. All statistical analyses were performed using R version 3.4.1 ( R Core Team, 2017), with the package *mice* utilised for multiple imputation ( Buuren and Groothuis-Oudshoorn, 2011).

## Results/Analysis

Data were obtained from those who enrolled between the first semester 2009 and second semester 2017, representing 1499 students of whom 192 were enrolled in the master’s programme. These students had registered on a total of 5596 modules, of which 2540 were completed (43.4% of all module registrations), and 2077 modules were passed (37.1% of all module registrations, 82.2% of those completed). 624 students have ever completed a module (41.6% of all students registered), 513 students have ever passed a module (34.2% of all students registered, 82.2% of those students who had ever completed a module). 503 students (33.6%) had applied for a bursary - of these, 285 (56.7% of those who applied and 19% of all students) were given a bursary.

Table 1 shows the demographic, education and employment characteristics of enrolled students. Almost two-thirds of the students were male and the median age at first registration was 36 years. The largest number of student registrations were from Nigeria (n=392) and India (n=129), with 71% of all students based in Africa. A third of students had a medical degree and just over a third worked in Public Health.

**Table 1.** General characteristics of registered students. (Total n=1499)

**Sex** (n=1456)		n	%
	Male	917	63.0%
	Female	539	37.0%
**Age** (n=1451)		**mean**	**s.d.**
	years	35.7	8.0
**Region** (n=1499)		**n**	**%**
	Africa	1065	71.0%
	Asia	291	19.4%
	Europe	87	5.8%
	North America	36	2.4%
	South America	12	0.8%
	Oceania	8	0.5%
**Qualification Type** (n=1499)		**n**	**%**
	Degree - medical	492	32.8%
	Degree - other health	472	31.5%
	Degree - not health	351	23.4%
	Non-degree health qualification	151	10.1%
	None	33	2.2%
**Employment** (n=1499)		**n**	**%**
	Public Health	590	39.4%
	Clinical, not public health	367	24.5%
	Other health-related	250	16.7%
	Not health-related	56	3.7%
	Academic	113	7.5%
	Student	46	3.1%
	None	77	5.1%

Compared with those who did not apply for a bursary, bursary applicants (successful or otherwise) were more likely to be male and to have registered for the master’s programme ( Table 2). Successful applicants were statistically significantly more likely to have come from Africa and less likely to have a medical degree than other students.

**Table 2.** Characteristics of students who received and did not receive bursaries

		Non-applicants (n=996)	Unsuccessful applicants (n=218)	Successful applicants (n=285)	
**Male**		59%	72%	71%	p < 0.001
**Age (mean, s.d.)**		35.5 (8.1)years	36.8 (8.4) years	35.6 (7.6) years	p = 0.103
**Region**	Africa	68%	71%	81%	p < 0.001
	Asia	20%	21%	16%	
	Europe	8%	3.2%	1.4%	
	North America	2.5%	3.7%	1%	
	South America	0.9%	1.4%	0%	
	Oceania	0.6%	0.5%	0.3%	
**Qualification**	Degree - medical	35%	31%	25%	p = 0.020
	Degree - other health	30%	30%	37%	
	Degree - not health	23%	26%	22%	
	Non-degree health qualification	9%	11%	9%	
	None	2%	2%	2%	
**Employment**	Public Health	39%	44%	36%	p = 0.589
	Clinical, not public health	25%	22%	23%	
	Other health-related	17%	13%	19%	
	Not health-related	4%	4%	4%	
	Academic	8%	6%	9%	
	Student	3%	5%	4%	
	None	5%	6%	6%	
**Master’s registration**		7%	27%	23%	p < 0.001

Table 3 shows the factors associated with successfully receiving a bursary. In both unadjusted and adjusted analyses, the following factors were found to be independently associated with having received a bursary: male sex (adjusted odds ratio [aOR] 1.5, 95% confidence interval [CI] 1.1,2.0); residing in Africa (aOR 6.1, 95% C 2.4,20.5); residing in Asia (aOR 4.7, 95% CI 1.8,16.5); having an ‘other (non-medical) health degree’ (aOR 1.8, 95% CI 1.2,2.5); having an ‘other health qualification’ (aOR 2.0, 95% CI 1.3,3.3); and having enrolled in the master’s programme (aOR 2.7, 95% CI 1.9,3.9).

**Table 3.** Factors associated with receiving a bursary. (†adjusted for all variables presented)

		Unadjusted		†Adjusted	
		Odds Ratio (95% CI)	p value	Odds Ratio (95% CI)	p value
**Age**	(per 10 year age increase)	1.0 (1.0,1.0)	p = 0.268	1.0 (1.0,1.0)	p = 0.258
**Sex**	Female	Reference		Reference	
	Male	1.5 (1.1,2.0)	p = 0.004	1.5 (1.1,2.0)	p = 0.009
**Region**	Europe	Reference		Reference	
	Africa	5.7 (2.4,19.0)	p < 0.001	6.1 (2.4,20.7)	p < 0.001
	Asia	3.8 (1.5,12.9)	p = 0.013	4.7 (1.8,16.5)	p = 0.005
	North America	1.9 (0.4,9.0)	p = 0.422	2.1 (0.8,10.2)	p = 0.362
	South America	(no bursaries received)		(no bursaries received)	
	Oceania	3.0 (0.1,23.8)	p = 0.359	3.2 (0.2,27.1)	p = 0.332
**Qualification**	Medical degree	Reference		Reference	
	Other health degree	1.7 (1.2,2.4)	p = 0.002	1.8 (1.2,2.5)	p = 0.002
	Other health qualification	2.1 (1.4,3.3)	p < 0.001	2.0 (1.3,3.3)	p = 0.003
	Non-health degree	1.3 (0.9,1.9)	p = 0.138	1.4 (0.9,2.2)	p = 0.084
	None	1.1 (0.4,2.6)	p = 0.909	1.3 (0.4,3.3)	p = 0.651
**Employment**	Public health	Reference		Reference	
	Clinical (not public health)	1.0 (0.7,1.4)	p = 0.888	1.1 (0.8,1.6)	p = 0.491
	Other health-related	1.3 (0.9,1.8)	p = 0.225	1.3 (0.9,1.9)	p = 0.207
	Non-health	1.0 (0.5,2.0)	p = 0.966	1.1 (0.5,2.3)	p = 0.746
	Academic	1.3 (0.8,2.1)	p = 0.259	1.4 (0.8,2.3)	p = 0.195
	Student	1.5 (0.7,2.9)	p = 0.289	1.9 (0.9,3.8)	p = 0.095
	None	1.2 (0.7,2.2)	p = 0.499	1.8 (0.9,3.2)	p = 0.076
**M** **aster’s** **Registration**		2.6 (1.9,3.6)	p < 0.001	2.7 (1.9,3.9)	p < 0.001

Students with a successful bursary application had a higher odds of ever completing a module, compared to never-applicants (aOR 2.7, 95% CI 2.0,3.7, Table 4). Students who were unsuccessful in their bursary applications were also more likely to complete a module (aOR 1.8, 95% CI 1.3,2.6). Other factors positively associated with ever completing a module included increasing age and master’s registration, whilst males were less likely to complete a module (aOR 0.7, 95% CI 0.5,0.9).

In line with the findings above, compared to never-applicants, students with a successful bursary application had a higher odds of ever passing a module, (aOR 2.3, 95% CI 1.7,3.3, Table 5), as did those who applied unsuccessfully (aOR 1.9, 95% CI 1.3,2.8). Both increasing age and master’s registration were positively associated with ever passing a module, and males were less likely to ever pass a module.

Success or failure of bursary applicants was not independently associated with ever completing a module (aOR 1.4, 95% CI 0.9,2.2, p=0.121, successful *vs.* unsuccessful applicants). Similarly, success or failure of bursary applicants was not independently associated with ever passing a module (aOR 1.1, 95% CI 0.7,1.8, p=0.577, successful *vs.* unsuccessful applicants).

**Table 4.** Factors associated with ever completing a module. (†adjusted for all variables presented)

		Unadjusted		Adjusted†	
		Odds Ratio (95% CI)	p value	Odds Ratio (95% CI)	p value
**Age**	(per 10 year age increase)	1.0 (1.0,1.0)	p = 0.040	1.0 (1.0,1.0)	p = 0.002
**Sex**	Female	Reference		Reference	
	Male	0.8 (0.7,1.0)	p = 0.096	0.7 (0.5,0.9)	p = 0.001
**Region**	Europe	Reference		Reference	
	Africa	1.0 (0.6,1.6)	p = 0.999	0.8 (0.5,1.4)	p = 0.527
	Asia	0.8 (0.5,1.3)	p = 0.332	0.6 (0.3,1.1)	p = 0.077
	North America	1.5 (0.7,3.3)	p = 0.300	1.3 (0.5,3.1)	p = 0.558
	South America	1.0 (0.3,3.3)	p = 0.955	0.3 (0.0,1.6)	p = 0.213
	Oceania	0.8 (0.2,3.5)	p = 0.783	1.1 (0.2,5.2)	p = 0.863
**Qualification**	Medical degree	Reference		Reference	
	Other health degree	1.0 (0.8,1.3)	p = 0.972	0.9 (0.6,1.2)	p = 0.408
	Other health qualification	0.9 (0.6,1.4)	p = 0.724	0.7 (0.4,1.1)	p = 0.121
	Non-health degree	0.9 (0.7,1.2)	p = 0.371	0.8 (0.6,1.2)	p = 0.271
	None	0.7 (0.3,1.4)	p = 0.295	0.6 (0.2,1.3)	p = 0.224
**Employment**	Public health	Reference		Reference	
	Clinical (not public health)	1.0 (0.8,1.3)	p = 0.974	0.9 (0.7,1.3)	p = 0.661
	Other health-related	1.0 (0.7,1.4)	p = 0.984	1.0 (0.7,1.4)	p = 0.996
	Non-health	0.8 (0.4,1.4)	p = 0.399	0.8 (0.4,1.6)	p = 0.618
	Academic	1.2 (0.8,1.7)	p = 0.477	1.0 (0.6,1.6)	p = 0.992
	Student	1.3 (0.7,2.4)	p = 0.405	1.3 (0.6,2.5)	p = 0.472
	None	0.9 (0.5,1.5)	p = 0.667	0.7 (0.4,1.2)	p = 0.155
**M** **aster’s** **Registration**		191 (61,1158)	p < 0.001	172 (54,1050)	p < 0.001
**Bursary**	Non-applicant	Reference		Reference	
	Unsuccessful applicant	2.6 (1.9,3.5)	p < 0.001	1.8 (1.3,2.6)	p < 0.001
	Successful applicant	3.1 (2.4,4.1)	p < 0.001	2.7 (2.0,3.7)	p < 0.001

Note: Odds ratios reported to one decimal place. For age,with each 10-year increase in student age, there is a 1.00041 increased odds of completing a module (95% CI 1.00002,1.00081).

**Table 5.** Factors associated with ever passing a module. (†adjusted for all variables presented)

		Unadjusted		Adjusted†	
		Odds Ratio (95% CI)	p value	Odds Ratio (95% CI)	p value
**Age**	(per 10 year age increase)	1.0 (1.0,1.0)	p = 0.163	1.0 (1.0,1.0)	p = 0.007
**Sex**	Female	Reference		Reference	
	Male	0.8 (0.6,0.9)	p = 0.014	0.6 (0.4,0.7)	p < 0.001
**Region**	Europe	Reference		Reference	
	Africa	0.9 (0.6,1.5)	p = 0.756	0.8 (0.5,1.4)	p = 0.403
	Asia	0.7 (0.4,1.1)	p = 0.111	0.4 (0.2,0.8)	p = 0.008
	North America	1.5 (0.7,3.4)	p = 0.283	1.3 (0.5,3.2)	p = 0.580
	South America	0.9 (0.2,3.0)	p = 0.816	0.1 (0.0,0.9)	p = 0.084
	Oceania	0.6 (0.1,2.7)	p = 0.510	0.8 (0.1,4.2)	p = 0.851
**Qualification**	Medical degree	Reference		Reference	
	Other health degree	1.0 (0.7,1.2)	p = 0.739	0.8 (0.6,1.1)	p = 0.211
	Other health qualification	0.9 (0.6,1.3)	p = 0.549	0.6 (0.4,1.0)	p = 0.048
	Non-health degree	0.9 (0.6,1.1)	p = 0.281	0.8 (0.5,1.2)	p = 0.253
	None	0.8 (0.3,1.6)	p = 0.526	0.7 (0.3,1.7)	p = 0.426
**Employment**	Public health	Reference		Reference	
	Clinical (not public health)	1.0 (0.8,1.3)	p = 0.981	0.9 (0.6,1.3)	p = 0.549
	Other health-related	1.0 (0.7,1.3)	p = 0.822	1.0 (0.7,1.4)	p = 0.813
	Non-health	0.6 (0.3,1.2)	p = 0.157	0.6 (0.3,1.3)	p = 0.200
	Academic	1.2 (0.8,1.8)	p = 0.356	1.1 (0.6,1.8)	p = 0.813
	Student	1.3 (0.7,2.5)	p = 0.346	1.4 (0.7,2.9)	p = 0.301
	None	0.8 (0.5,1.3)	p = 0.430	0.5 (0.3,1.0)	p = 0.054
**Master’s Registration**		289 (92,1754)	p < 0.001	306 (93,1990)	p < 0.001
**Bursary**	Non-applicant	Reference		Reference	
	Unsuccessful applicant	2.7 (2.0,3.7)	p < 0.001	1.9 (1.3,2.8)	p < 0.001
	Successful applicant	2.8 (2.2,3.7)	p < 0.001	2.3 (1.7,3.3)	p < 0.001

Note: Odds ratios reported to one decimal place. For age,with each 10-year increase in student age, there is a 1.00061 increased odds of completing a module (95% CI 1.00017,1.00104).

## Discussion

Around 20% of students were given a bursary, and students who received a bursary had around twice the odds of ever completing, and ever passing a module, when adjusted for all other measured variables. Similar results were obtained for those who had applied unsuccessfully for a bursary. As expected, being enrolled in the master’s programme was strongly positively associated with completing and passing, since passing two modules was a requirement for such enrolment. Males were less likely to complete and pass a module than females, and older students were more likely than younger students to complete and pass a module. However the increase with age, while statistically significant was not of practical importance - with each 10-year increase in student age, there was only a 0.04% increase in odds of completing, and 0.06% increase in odds of passing, a module.

The results point to the success of the bursary scheme, since those who were given a bursary having been assessed by the bursary team to have genuine problems with paying the course fees not only completed and passed modules, but were more likely to do so than those who had not applied for a bursary. The unexpected finding that those whose application for a bursary was unsuccessful were also more likely to complete and pass leads us to conclude that the application for a bursary was an indication of the motivation to participate and succeed in the programme. That the bursary assessment team was able to identify those who did not in fact require a bursary to succeed, is a testament to the criteria they applied to the applications. The feedback process for the unsuccessful applicants, and ability to offer payments by installments might also have contributed to the continued involvement of those refused a bursary. Those who are successful in getting a bursary may have done so because the panel who make the decision judge them to have a more convincing reason for needing the bursary and/or for using the skills gained in the programme. Additionally, successful bursary applicants may feel a sense of responsibility to successfully complete modules, given that they have been subsidised. Of those applicants who do not get a bursary, some may choose not to study as they either can’t afford it or because the personal cost/motivation equation leans towards it not being worth it, and this group are thus not included in the analysis. Those failed bursary applicants that still decide to study and pay their own way would have reasonable motivation to remain committed so that their investment isn’t wasted. This latter reason may not apply to those who never applied for a bursary as it may not be such an issue for them if their course fees were lost. An alternative interpretation, in view of the lack of difference between the successful and unsuccessful applicants, is that the bursary scheme was a confounder for some other predictor of success and just identified those who wanted to reduce the costs of study.

An earlier examination of pass rates in People-uni, prior to the introduction of the master’s programme with many fewer numbers and without the use of multivariate analysis, found that those who paid their fees were more likely to pass than those who did not ( Philip and Lee, 2011). They reported that engagement with the course was the major predictor of passing.

A study from three Australian universities found limited and inconsistent effects of scholarships on student outcome ( Zacharias *et al.*, 2016). A study of the efficacy of a scholarship program designed to assist single-parent, full-time undergraduate students found that single parents who participated in the scholarship program had higher levels of academic achievement, degree completion rates, and greater progress toward completion than non-participants ( Carpenter *et al.*, 2018). Evaluations of the impact of two other scholarship programmes for students from disadvantaged backgrounds in Australia found improved retention rates ( Carson, 2010; Reed and Hurd, 2016). Despite the fact that many universities offer scholarships or bursaries, the literature on the outcomes is sparse. We believe that our findings add substantially to the literature and may be of use to others.

There is considerable literature on motivation as a predictor of academic success, for example ( Guiffrida *et al.*, 2013) reported that students who were motivated to attend college to fulfill intrinsic needs for autonomy and competence gained higher grades and demonstrated greater intentions to persist than students who were less motivated to attend for these reasons.

### Study limitations

The numbers reported here are slightly different from those reported in our recent paper on student characteristics covering a similar period ( Heller, Strobl and Madhok, 2019). We have restricted this analysis to those in whom we have full availability of demographic and outcome data for the relevant semesters. By exploring the outcome by students ever or never receiving a bursary we have lost granularity in the analysis, while reducing its complexity. Students can either enter the course to study individual modules, or with the aim of gaining a diploma or master’s award, and might apply for a bursary in some semesters only, and accounting for all these factors simultaneously would involve a complex analysis and limit interpretability. The decision to award a bursary in those who apply in multiple semesters might have been influenced by academic success in earlier semesters, but analysis at the student rather than module or semester level will have reduced the likelihood of any potential bias from this cause. Such a bias would only have existed between successful and unsuccessful applicants rather than between applicants and non-applicants. We have not explored the outcome of gaining the master’s award, as a number of the students are still in the process of working through the course, but instead we examined enrolment in the master’s programme. Although the questions asked on the bursary application form are reproducible, the adjudication process was not standardised and might not give similar results in different settings and with different adjudicators. We have not explored the reasons for the request for a bursary, although the data exist and could form the basis for further study.

## Conclusion

Despite the low fees charged in this volunteer-led programme, those who could make the case of need and receive a bursary were more likely to complete and pass modules than those who had not applied for a bursary. The literature on the outcome of scholarships programmes is sparse, and the results give us confidence to continue to offer bursaries to those in need, in order to sustain the mission of improving population health through capacity building in low resource settings.

## Take Home Messages

• In the context of an online master’s level programme to build Public Health capacity in low- to middle-income countries, those who were given a bursary to reduce or waive the fees were more likely to complete and pass course modules than those who did not apply for a bursary
• Those who applied unsuccessfully for a bursary were also more likely to complete and pass course modules than those who did not apply
• There is little published research on the outcome of bursaries to reduce student fees, but we are encouraged to continue our policy of inviting applications for, and awarding, bursaries

## Notes On Contributors

Dr Pasipanodya Ian Machingura, PhD, DPhil, MPH, is Chairman and Senior Lecturer, Department of Medical Laboratory Sciences, University of Zimbabwe, Zimbabwe. He is a graduate of Peoples-uni and research coordinator of the Alumni group

Dr Saran Shantikumar, MB ChB, PhD, is a clinical lecturer in public health at Warwick Medical School, University of Warwick, England. He is a tutor for Peoples-uni. Orcid reference https://orcid.org/0000-0002-8565-5385

Ms Olukemi Solabomi Babalola, MD, MSc, is Project Manager and Researcher, Center for Health Policy, University of the Witwatersrand, Johannesburg, South Africa. She is a tutor for Peoples-uni.

Professor Richard F Heller, MD, FRCP, FFPH, is Emeritus Professor, Universities of Newcastle Australia and Manchester UK, and Coordinator and Trustee of the Peoples-uni. Orcid reference https://orcid.org/0000-0003-3161-5967
