# Supplementary material for: Does a bursary scheme for students in low- to middle-income countries influence outcomes in a master’s programme in Public Health?
Source: MedEdPublish (2016). 2019 Jun 4;8:122. [Version 1] doi: 10.15694/mep.2019.000122.1 (PMC10712521; doi:10.15694/mep.2019.000122.1)
Supplement: Peer review history

# Reviewer report: Reviewer response for version 1

**DOI:** 10.21956/mep.19855.r29972

**Published:** 2019-07-27

**Authors:** Judy McKimm

**Affiliations:** 1 Swansea University

This review has been migrated. The reviewer awarded 4 stars out of 5 I enjoyed reading this paper and I thought that the somewhat unanticipated finding that unsuccessful bursary applicant also succeeded well was interesting. Like Trevor, I would like to see more qualitative research on this regarding the motivating factors and follow up of career development and impact. I would have liked to know more about the organisation and countries involved at the start , I wouldn't think this would be too revealing but it would certainly help readers understand the context ion which this operates.

Reviewer Expertise:

NA

No decision status is available

# Reviewer report: Reviewer response for version 1

**DOI:** 10.21956/mep.19855.r29975

**Published:** 2019-07-07

**Authors:** Trevor Gibbs

**Affiliations:** 1 AMEE

This review has been migrated. The reviewer awarded 4 stars out of 5 As someone who spends most of his teaching time in resource constrained countries, I enjoyed reading this paper. Although I think that the outcomes of the paper could have been predicted, it no doubt adds to our beliefs that reward and support systems are required to break through the finances barrier to education. My main concern would be the longevity of this effect, although I believe that the initial uplift provided is enough to increase the internal motivation of the students for future work.I would recommend this paper to anyone involved in developing and delivering such courses.I hope that the authors continue to study within this area to perhaps explore the longer term effects and perhaps, by using a more qualitative approach, look at the reasons why the students succeed with such a small financial uplift.

Reviewer Expertise:

NA

No decision status is available

# Reviewer report: Reviewer response for version 1

**DOI:** 10.21956/mep.19855.r29971

**Published:** 2019-07-07

**Authors:** Balakrishnan Nair

**Affiliations:** 1 Centre for Medical Professional Development and University of Newcastle

This review has been migrated. The reviewer awarded 4 stars out of 5 This is an interesting paper. It is good to see more than 40% completion rate in any online course. What is more interesting is that people who were successful in getting the bursaries had a higher completion rate. When one considers that the bursary was only 50 pounds , which is not huge amount, we need to pause to think about our colleagues in developing countries. The team running the program should be applauded for the work they do It will be good to see whether the candidates completed the Masters after obtaining the bursary

Reviewer Expertise:

NA

No decision status is available

# Reviewer report: Reviewer response for version 1

**DOI:** 10.21956/mep.19855.r29974

**Published:** 2019-06-05

**Authors:** Tan Nguyen

**Affiliations:** 1 Deakin University

This review has been migrated. The reviewer awarded 4 stars out of 5 This paper is worth a read and provides further insights into the study field. Further context and full description would be useful regarding the criteria for the bursary, as this in itself can be 'self-selecting'. For example, successful bursary recipients may be highly motivated candidates with the previous history of academic excellence. which could be associated with a higher odds ratio of module completions. It is important to also consider what is considered 'success' for the introduction of the bursary. i.e. is the bursary program designed to attract and retain candidates to achieve higher degree academic completions at Master's level, as this was not evident in the work.

Reviewer Expertise:

NA

No decision status is available

# Reviewer report: Reviewer response for version 1

**DOI:** 10.21956/mep.19855.r29973

**Published:** 2019-06-04

**Authors:** Robert Hash

**Affiliations:** 1 American Medical Association

This review has been migrated. The reviewer awarded 5 stars out of 5 An interesting read! It would be good to follow this up with a study on what the graduates (bursary/bursary denied vs. paid tuition) did with the degree, and whether it fit the mission of the bursary program. That is, was the program intended to primarily raise the education/income level of the population, or was it intended to recruit individuals who would serve communities in need after graduation regardless of income-generating potential, or both.

Reviewer Expertise:

NA

No decision status is available
